# Supplementary material for: Zn-Enhanced Asp-Rich Antimicrobial Peptides: N-Terminal Coordination by Zn(II) and Cu(II), Which Distinguishes Cu(II) Binding to Different Peptides
Source: Int J Mol Sci. 2021 Jun 28;22(13):6971. doi: 10.3390/ijms22136971 (PMC8267837; doi:10.3390/ijms22136971)
Supplement: Supplementary file 1 [file ijms-22-06971-s001.zip › ijms-1266826-supplementary.pdf]

Supplementary Information

Zn-enhanced Asp-rich antimicrobial peptides: N-terminal coordination by Zn(II) and Cu(II), which distinguishes Cu(II) binding to different peptides

A.

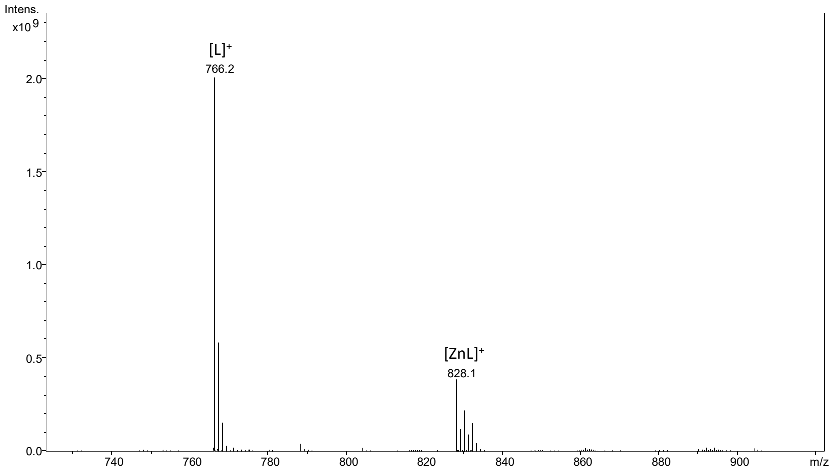

B.

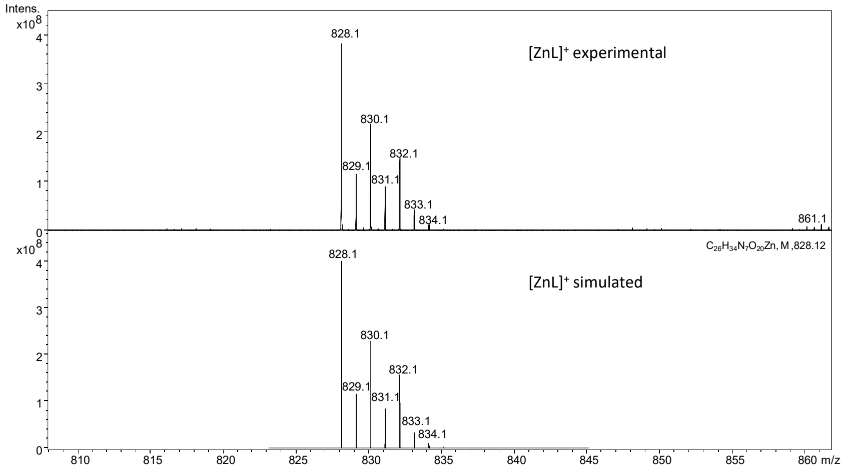

C.

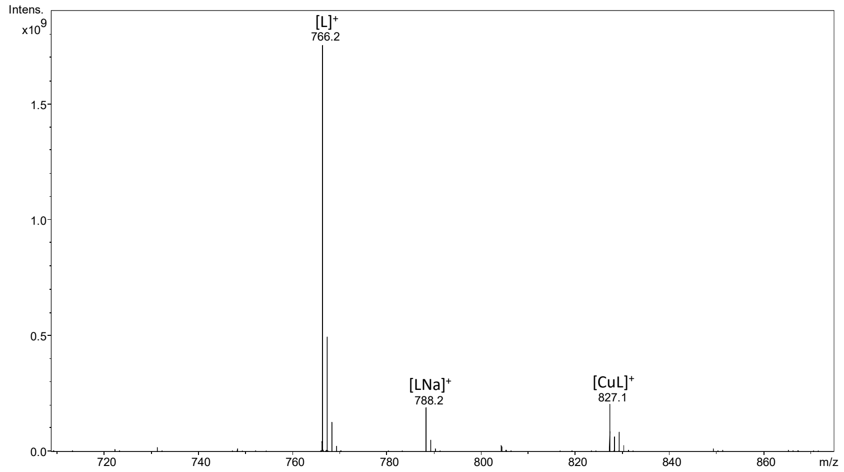
Intens.
x10^9
[L]⁺
766.2
[LNa]⁺
788.2
[CuL]⁺
827.1
1.5
1.0
0.5
0.0
720
740
760
780
800
820
840
860
m/z

D.

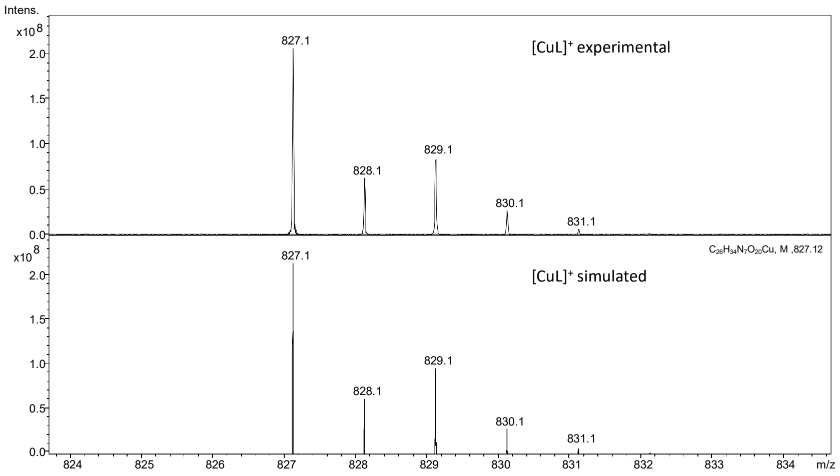
Intens.
x10^8
827.1
[CuL]⁺ experimental
828.1
829.1
830.1
831.1
2.0
1.5
1.0
0.5
0.0
x10^8
$C_{26}H_{34}N_7O_{20}Cu$, M ,827.12
827.1
[CuL]⁺ simulated
828.1
829.1
830.1
831.1
824
825
826
827
828
829
830
831
832
833
834
m/z

E.

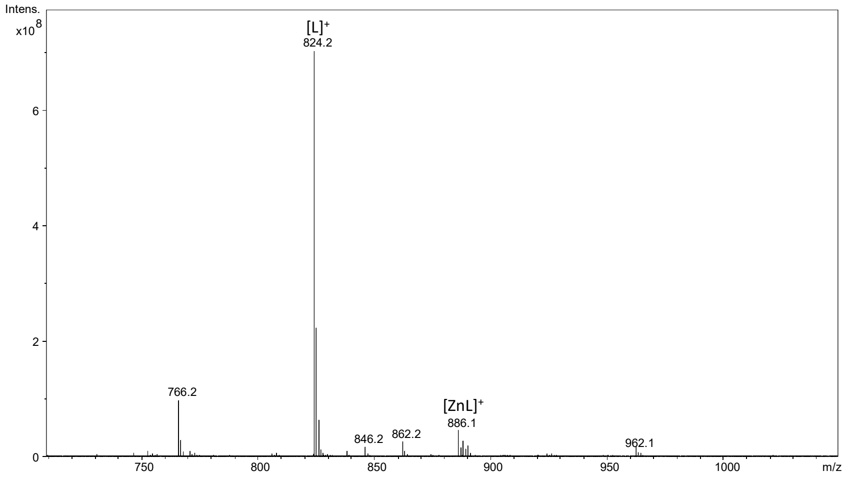
Intens.
x10^8
[L]⁺
824.2
6
4
2
0
766.2
846.2
862.2
[ZnL]⁺
886.1
962.1
750
800
850
900
950
1000
m/z

F.

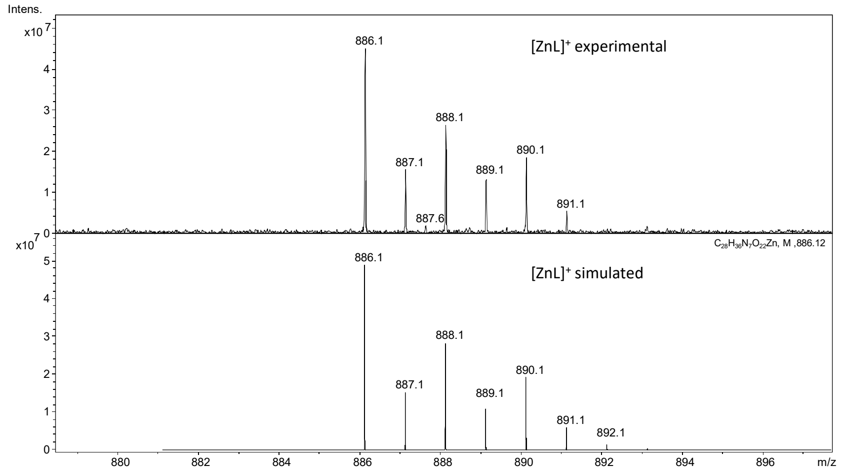
Intens.
x10^7
[ZnL]+ experimental
886.1
887.1
887.6
888.1
889.1
890.1
891.1
C28H36N7O22Zn, M ,886.12
[ZnL]+ simulated
892.1
m/z

G.

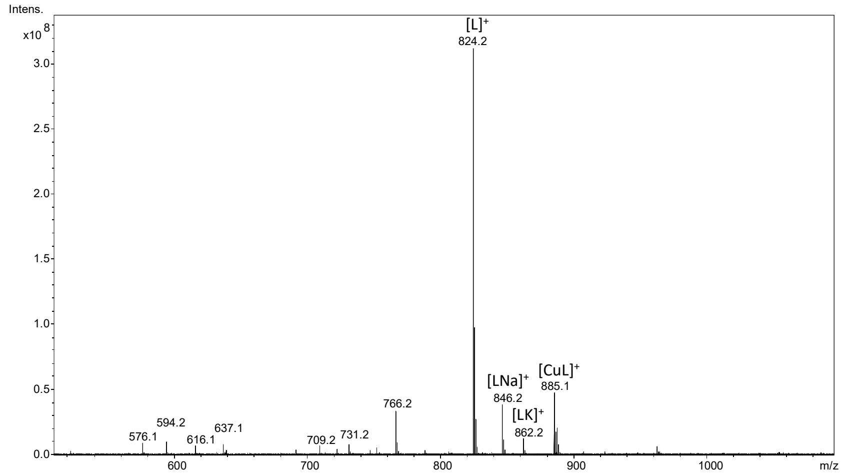
Intens.
x10^8
[L]+
824.2
576.1
594.2
616.1
637.1
709.2
731.2
766.2
[LNa]+
846.2
[LK]+
862.2
[CuL]+
885.1
m/z

H.

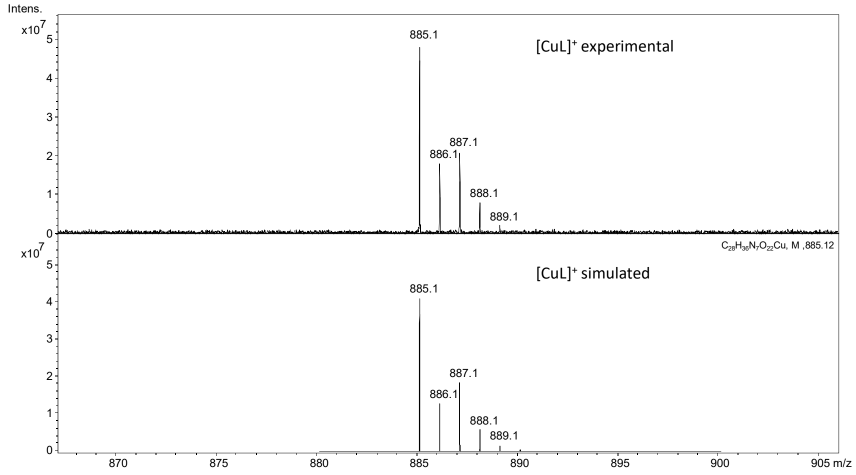
Intens.
x10^7
[CuL]+ experimental
885.1
886.1
887.1
888.1
889.1
C28H36N7O22Cu, M ,885.12
[CuL]+ simulated
m/z

I.

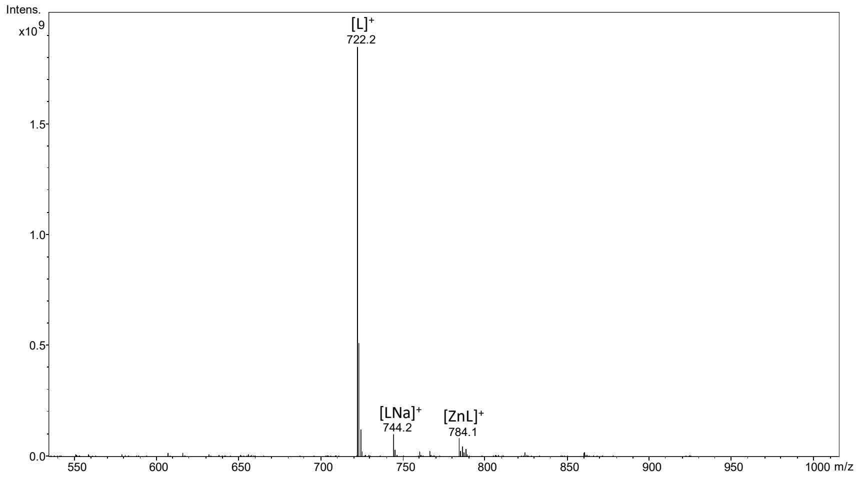

Intens.
x10^9
[L]+
722.2
[LNa]+
744.2
[ZnL]+
784.1
m/z

J.

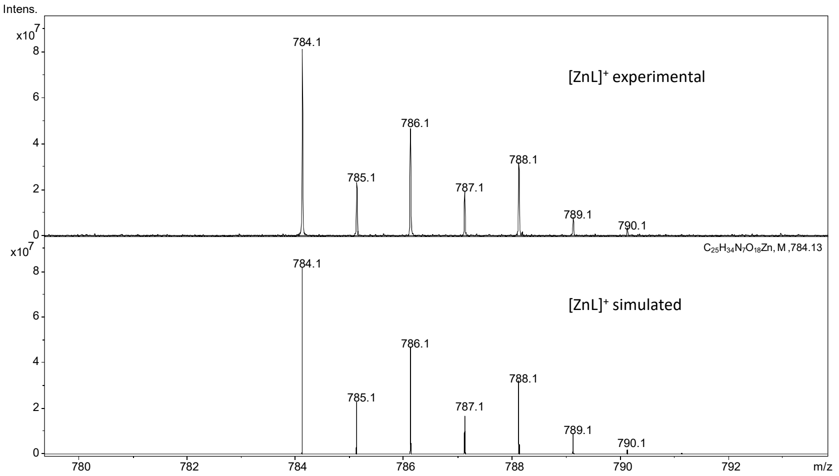

Intens.
x10^7
784.1
785.1
786.1
787.1
788.1
789.1
790.1
[ZnL]+ experimental
C25H34N7O18Zn, M ,784.13
[ZnL]+ simulated
m/z

K.

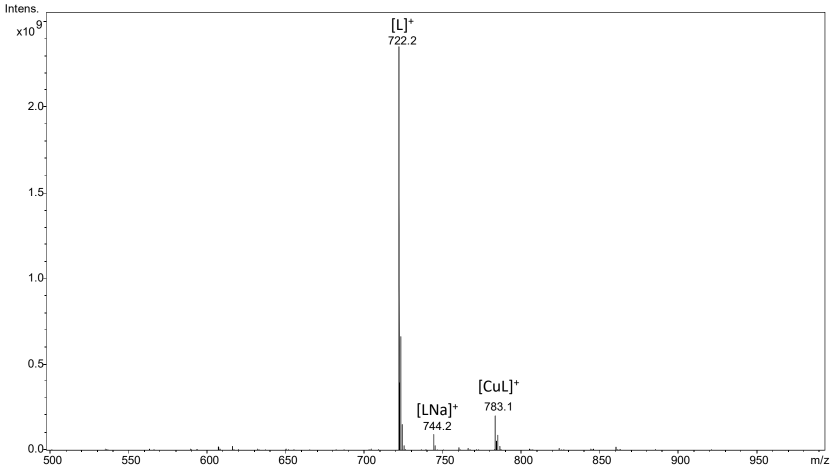

Intens.
x10^9
[L]+
722.2
[LNa]+
744.2
[CuL]+
783.1
m/z

L.

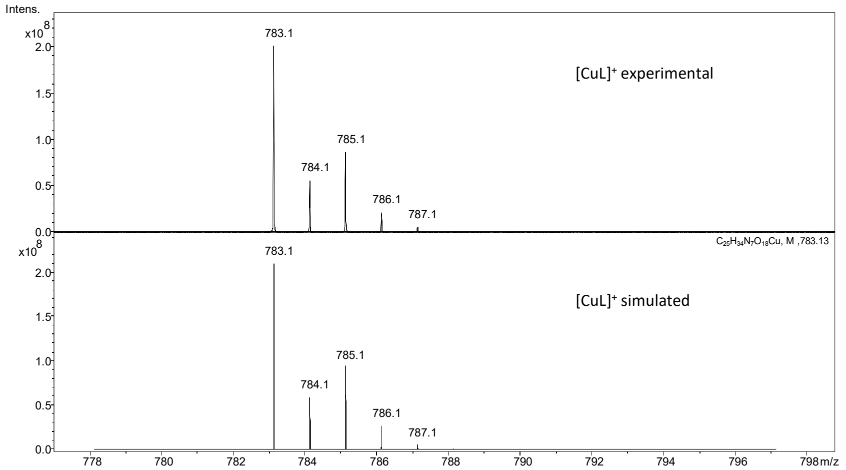

Figure S1 *MS spectra of Zn(II)-SAAP2 (A,B), Cu(II)-SAAP2 (C,D), Zn(II)-SAAP3 (E,F), Cu(II)-SAAP3 (G,H), Zn(II)-SAAP6 (I,J), Cu(II)-SAAP6 (K,L) samples. In the B, D, F, H, J, and L spectra, the experimental (top) and simulated (bottom) results are compared to clearly show the presence of the complex. Conditions: [Zn(II)] = [Cu(II)] = [SAAP2] = [SAAP3] = [SAAP6] = 3 x 10^{-4} M in a 1:1 methanol-water mixture; M^{2+}:peptide ratio was 1:1*

A.

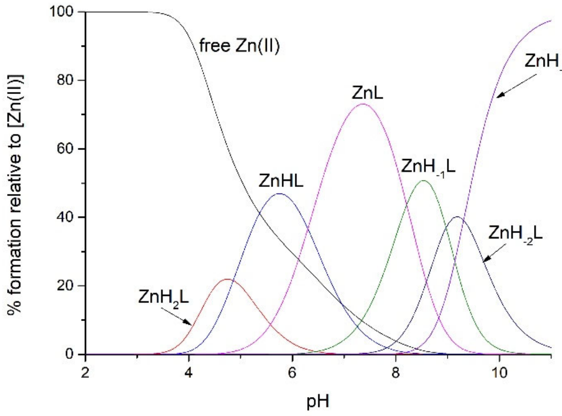

B.

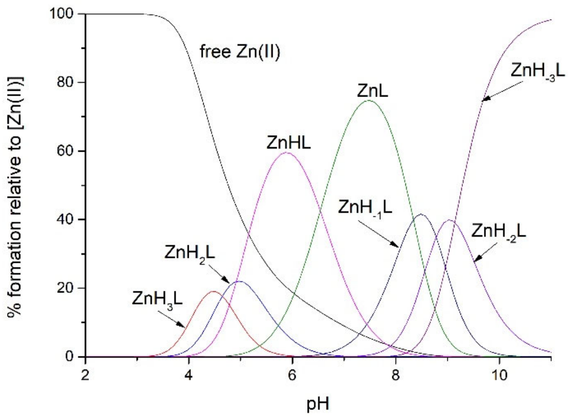

C.

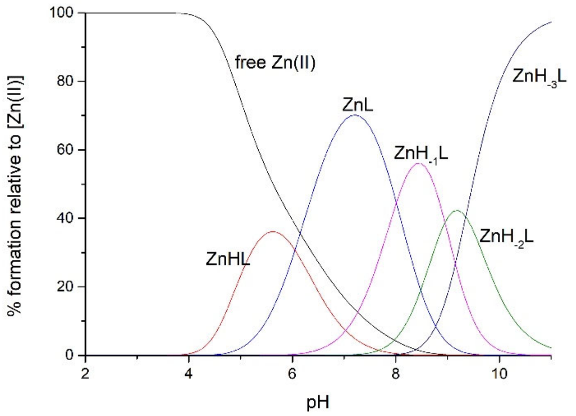

Figure S2 *Distribution diagrams for Zn(II) complexes with SAAP2 (A), SAAP3 (B) and SAAP6 (C); T = 298 K, 0.1 M NaClO$_4$, [Zn(II)] = 0.5 x 10^{-3} M; Zn(II):peptide molar ratio = 1:1*

A.

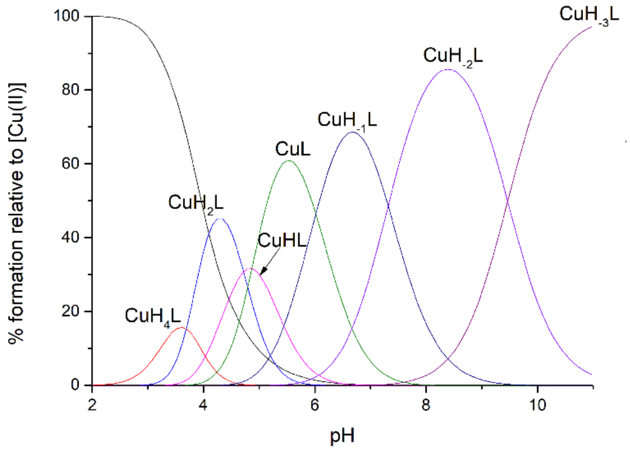

B.

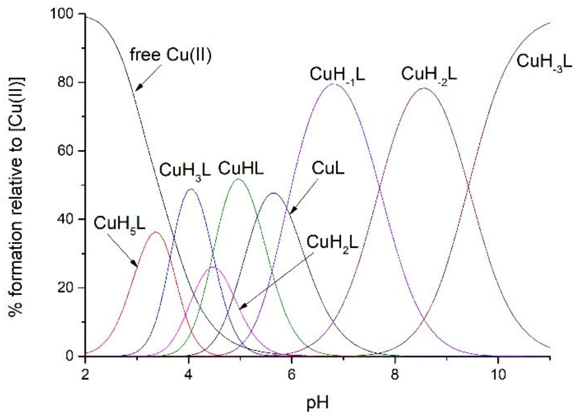

C.

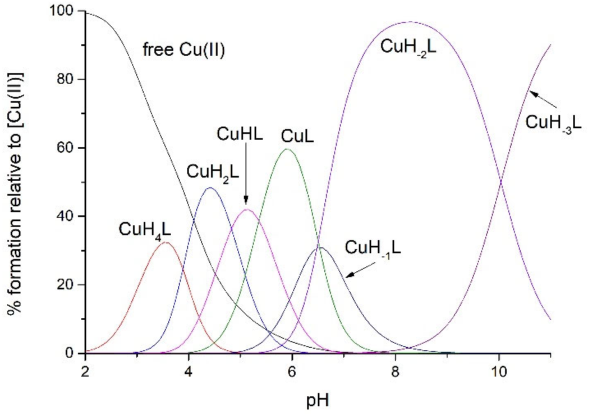

Figure S3 *Distribution diagrams for Cu(II) complexes with SAAP2 (A), SAAP3 (B) and SAAP6 (C); T = 298 K, 0.1 M $NaClO_4$, [Cu(II)] = 0.5 x 10^{-3} M; Cu(II):peptide molar ratio = 1:1*

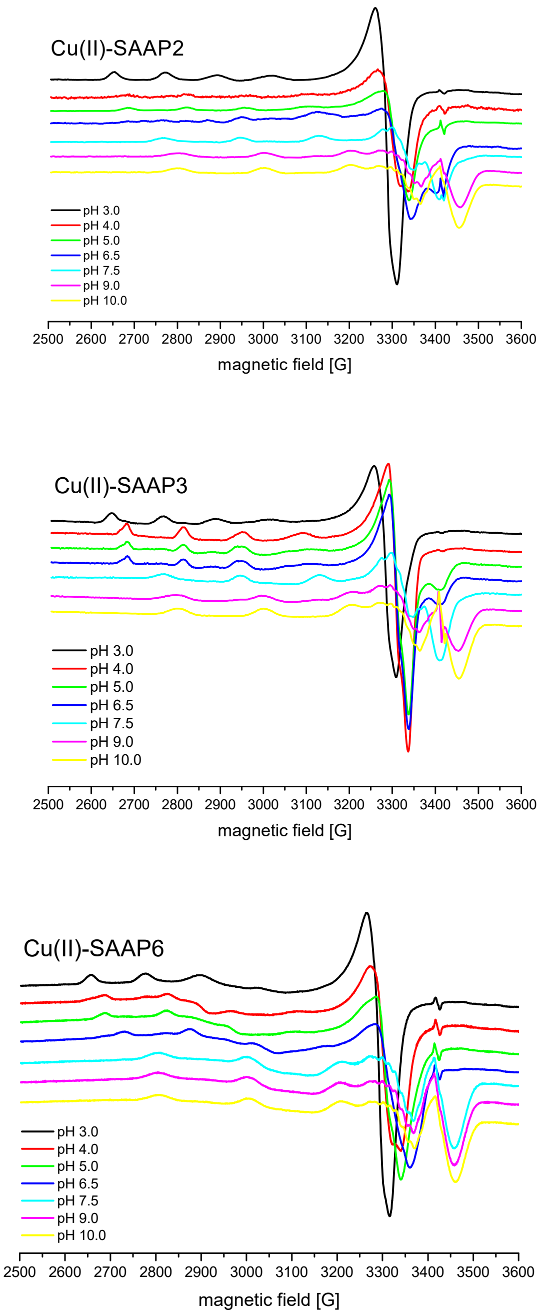

Figure S4 *X-band EPR spectra of frozen solutions (77 K) of Cu(II)-SAAP2, Cu(II)-SAAP3 and Cu(II)-SAAP6 at pH values between 3.0 and 10.0 (Cu(II)-peptide molar ratio = 1:1). Samples were in a buffered aqueous solution with 30% ethylene glycol as a cryoprotectant. Note, sharp signal at 3420 G is due to a contaminant in the cavity (see Figure S7).*

A.

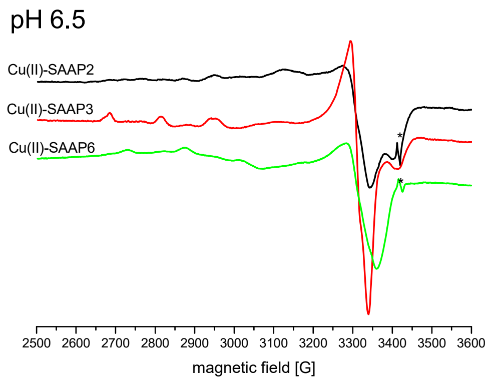

B.

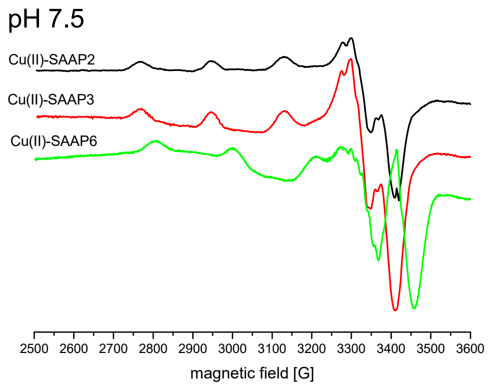

C.

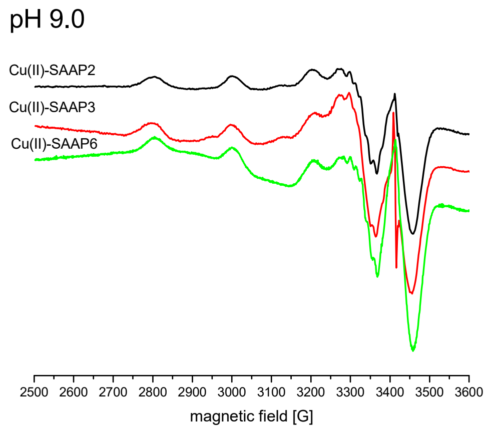

Figure S5 *Comparison of experimental X-band EPR spectra of frozen solutions (77 K) of Cu(II)-SAAP2 (black), Cu(II)-SAAP3 (red) and Cu(II)-SAAP6 (green) (Cu(II)-peptide molar ratio = 1:1) at pH 6.5 (A), 7.5 (B) and 9.0 (C). Samples were in a buffered aqueous solution with 30% ethylene glycol as a cryoprotectant. Note, sharp signal at 3420 G is due to a contaminant in the cavity (see Figure S7).*

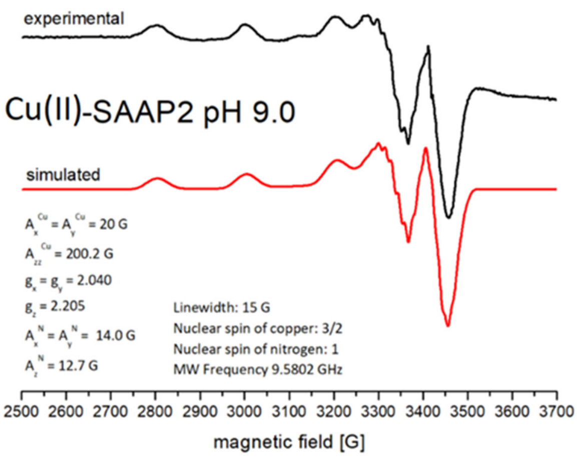

Figure S6 *Experimental (black) and simulated (red) EPR spectra of Cu(II)-SAAP2, confirming 4N coordination at pH 9. Similar simulation values were obtained for the analogous EPR spectra of Cu(II)-SAAP3 and Cu(II)-SAAP6.*

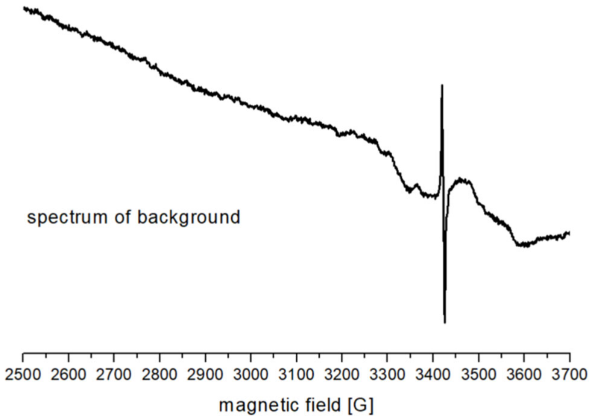

Figure S7 *The background spectrum obtained from the Bruker ELEXSYS E500 CW-EPR spectrometer before measurements of the Cu(II)-SAAP samples.*

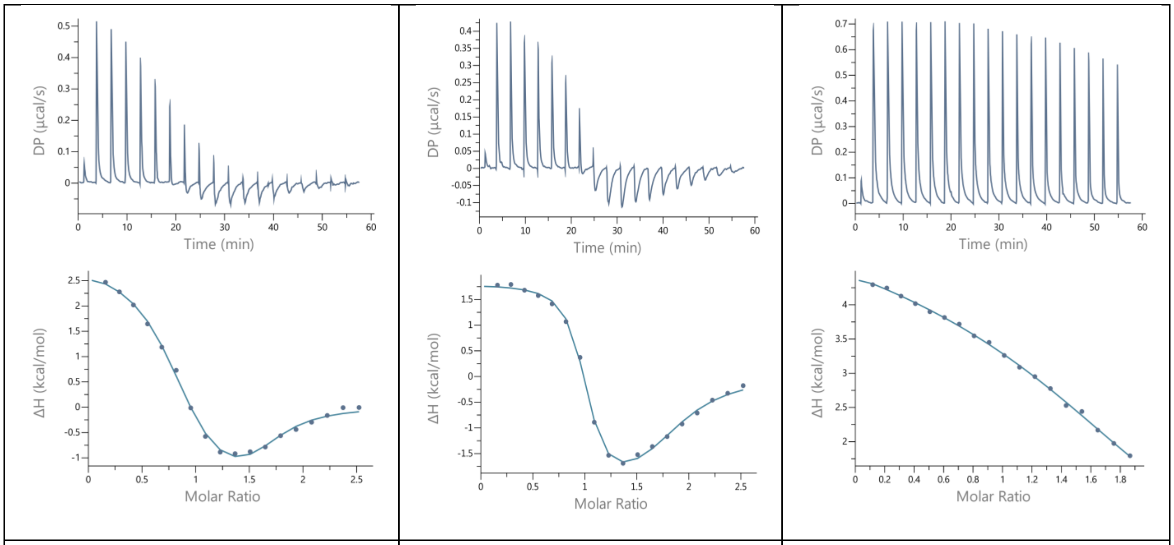

A) Cu(II) - SAAP2 | B) Cu(II) - SAAP3 | C) Cu(II) - SAAP6

Figure S8 *ITC titrations (raw data above; plots of integrated concentration-normalized data below) of 1.8-2 mM Cu(II) titrated into 150-200 µM of: A) SAAP2; B) SAAP3; and C) SAAP6 peptides (25 mM Caco buffer, pH 6.8).*

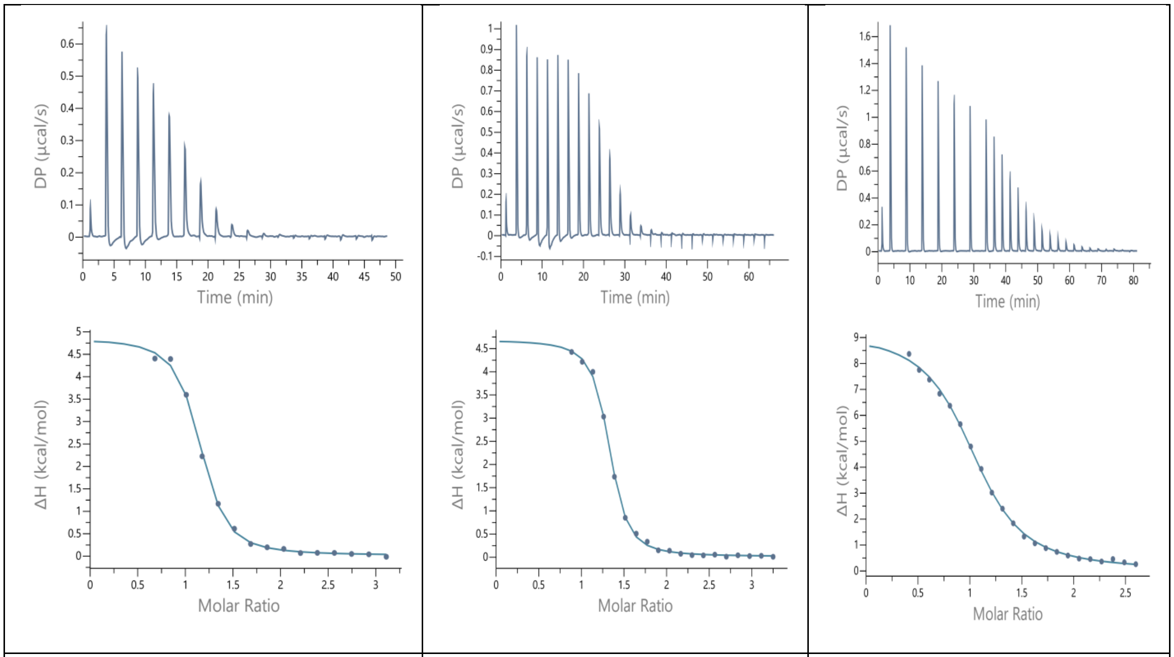

A) Cu(II) - SAAP2 | B) Cu(II) - SAAP3 | C) Cu(II) - SAAP6

Figure S9 *ITC titrations (raw data above; plots of integrated concentration-normalized data below) of A) SAAP2, B) SAAP3 and C) SAAP6 peptides titrated into solutions of Cu(II) ions (25 mM Caco buffer, pH 6.8).*
